# Detection of tuberculosis drug resistance: a comparison by *Mycobacterium tuberculosis* MLPA assay versus Genotype^®^MTBDR*plus*

**DOI:** 10.1590/0074-02760160376

**Published:** 2017-06

**Authors:** Paula Fernanda Gonçalves dos Santos, Elis Regina Dalla Costa, Daniela M Ramalho, Maria Lucia Rossetti, Regina Bones Barcellos, Luciana de Souza Nunes, Leonardo Souza Esteves, Rodrigo Rodenbusch, Richard M Anthony, Indra Bergval, Sarah Sengstake, Miguel Viveiros, Afrânio Kritski, Martha M Oliveira

**Affiliations:** 1 Universidade Federal do Rio de Janeiro, Faculdade de Medicina, Programa Acadêmico de Tuberculose, Programa de Pós-Graduação em Clínica Médica, Rio de Janeiro, RJ, Brasil; 2 Fundação Estadual de Produção e Pesquisa em Saúde, Centro de Desenvolvimento Científico e Tecnológico, Porto Alegre, RS, Brasil; 3 Universidade Luterana do Brasil, Porto Alegre, RS, Brasil; 4 Universidade Federal do Rio Grande do Sul, Centro de Biotecnologia, Programa de Pós-Graduação em Biologia Celular e Molecular, Porto Alegre, RS, Brasil; 5 Royal Tropical Institute, KIT Biomedical Research, Amsterdam, The Netherlands; 6 Universidade Nova de Lisboa, Instituto de Higiene e Medicina Tropical, Unidade de Microbiologia Médica, Global Health and Tropical Medicine, Lisboa, Portugal; 7 Rede Brasileira de Pesquisa em Tuberculose, Rio de Janeiro, RJ, Brasil

**Keywords:** tuberculosis, MLPA, GenoTypeMTBDR^®^*plus*, isoniazid resistance, rifampicin resistance, multidrug resistance

## Abstract

**BACKGROUND:**

To cope with the emergence of multidrug-resistant tuberculosis (MDR-TB), new molecular methods that can routinely be used to screen for a wide range of drug resistance related genetic markers in the *Mycobacterium tuberculosis* genome are urgently needed.

**OBJECTIVE:**

To evaluate the performance of multiplex ligaton-dependent probe amplification (MLPA) against Genotype® MTBDR*plus* to detect resistance to isoniazid (INHr) and rifampicin (RIFr).

**METHOD:**

96 culture isolates characterised for identification, drug susceptibility testing (DST) and sequencing of *rpo*B, *kat*G, and *inh*A genes were evaluated by the MLPA and Genotype®*MTBDRplus* assays.

**RESULTS:**

With sequencing as a reference standard, sensitivity (SE) to detect INHr was 92.8% and 85.7%, and specificity (SP) was 100% and 97.5%, for MLPA and Genotype®*MTBDRplus*, respectively. In relation to RIFr, SE was 87.5% and 100%, and SP was 100% and 98.8%, respectively. Kappa value was identical between Genotype®*MTBDRplus* and MLPA compared with the standard DST and sequencing for detection of INHr [0.83 (0.75-0.91)] and RIFr [0.93 (0.88-0.98)].

**CONCLUSION:**

Compared to Genotype®*MTBDRplus*, MLPA showed similar sensitivity to detect INH and RIF resistance. The results obtained by the MLPA and Genotype®*MTBDRplus* assays indicate that both molecular tests can be used for the rapid detection of drug-resistant TB with high accuracy. MLPA has the added value of providing information on the circulating *M. tuberculosis* lineages.

Tuberculosis (TB), despite being a curable infectious disease, persists as a global health problem that affects millions of people each year (WHO 2015). Drug-resistant and multidrug-resistant tuberculosis (DR/MDR-TB) have increased worldwide, demanding the development of new drug resistance detection assays. The early detection of mycobacterial clades can also support timely patient management, as some spoligofamilies have been associated with outbreaks, multidrug resistance or more aggressive forms of TB disease (Narvskaya et al. 2002, Gomes et al. 2012, Dalla Costa et al. 2015).

In some metropolitan Brazilian regions, increased rates of treatment default associated with increased rates of MDR-TB cases have been observed (Micheletti et al. 2014). As effective control of TB and MDR-TB is based on the rapid detection of *Mycobacterium tuberculosis* and drug resistance followed by appropriate and effective treatment, innovative and rapid diagnostic approaches and methods are urgently needed. This is especially true for difficult to manage TB cases, such as recurrence cases, relapse, treatment dropout or MDR suspicion, to ensure rapid and correct treatment decisions. Current molecular methods based on nucleic acid amplification allow identification of clustered genomic mutations and single nucleotide polymorphisms (SNPs) associated with drug resistance, particularly resistance to rifampicin (RIF) and/or isoniazid (INH); at present the two most important drugs of the standard first line treatment for TB (Steingart et al. 2014). The Genotype®*MTBDRplus* assay has been validated and it is currently in use in several countries, providing appropriate results for drug resistance detection (Schouten et al. 2002, Simons et al. 2015). However, no epidemiological information on the *M. tuberculosis* lineage is provided by this test. Thus, assays able to test for genome dispersed molecular characteristics in a single step are appealing and the multiplex ligation-dependent probe amplification (MLPA) technique is able to achieve the above-mentioned objective (Bergval et al. 2008). MLPA is a molecular method that allows simultaneous multiplex detection of a large number of SNPs and/or genetic markers by amplification of sequence-specific MLPA probes rather than the target DNA, without loss of amplification efficiency, as observed in a regular multiplex polymerase chain reaction (PCR) with too many amplification targets (Schouten et al. 2002). The presence of an MLPA product indicates the presence of the corresponding targeted SNP or genetic marker.

This study evaluates the ability of a highly-multiplexed MLPA assay designed to detect an extended number of mutations in different *M. tuberculosis* lineages isolated from Brazilian patients (Bergval et al. 2008). This MLPA assay was compared with the WHO-endorsed Genotype®*MTBDRplus* assay, using DNA sequencing and conventional drug susceptibility testing (DST) as reference gold-standard methods for the genotypic and phenotypic characterisation of drug resistance in these strains.

## MATERIALS AND METHODS

*Setting and routine laboratory procedures* - In a retrospective study carried out at a reference TB hospital and secondary Health Unit in the Rio de Janeiro city, Brazil, between January 2004 and July 2006, 96 *M. tuberculosis* isolates were obtained (one sample per patient) on the basis of routine testing. The isolates were confirmed as containing acid-fast bacilli by microscopy detection on Ziehl-Neelsen stained slides prepared from the cultures in Lowenstein-Jensen medium. Standard bacteriological and biochemical tests were performed for differentiation of species within the *M. tuberculosis* complex (MTBC) and mycobacteria other than tuberculosis (MOTT), including biochemical testing for niacin, paranitrobenoic acid (PNB) and tiofeno-2-carboxylic acid hydrazine (TCH) (MS 2010). Subsequently, the isolates were submitted to DST using the proportion method on Lowenstein-Jensen solid media (Canetti et al. 1969), following the Brazilian Ministry of Health recommended breakpoints for *M. tuberculosis* DST on solid media [isoniazid 0,2 µg/mL; ethambutol (EMB) 2 µg/mL; streptomycin 4 µg/mL; pyrazinamide (PZA) 25 µg/mL] (MS 2010).

*MLPA design of probes* - The probes used were as previously published and described by Bergval et al (2008), with exception the IS6110 probe. In summary: seventeen discriminatory markers were selected, and MLPA probes were designed accordingly, to provide information about drug resistance, principal genotypic group (PGG), and (mycobacterial) species (Table I) (Bergval et al. 2008). (i) Drug resistance markers (targeted by probes rpoB-522, rpoB-526G, rpoB- 526T, rpoB-531, rpoB-176, inhA-15, katG-315, and embB-306). Probe embB-306 targets the wild-type sequence, since many different base pair changes can occur in this codon. (ii) Genotypic markers (*gyr*A codon 95, *kat*G codon 463) to discriminate between the three PGGs. (iii) The mutT2 codon 58, mutT4 codon 48, ogt -12, ogt-37 and ogt-15 to identify putative virulent strains, such as the various Beijing and Haarlem lineages, respectively (Table I). (iv) Discriminatory region (specific to members of the MTBC): 16S rRNA gene. (v) Probe targeting TbD1: a region that is absent in the genome of “modern” *M. tuberculosis* strains but present in all other members of the MTBC.

**TABLE I:** Summary of the multiplex ligaton-dependent probe amplification (MLPA) probes used in this study as published by Bergval et al. (2008)

Probe	Target-specific sequence	Length (bp)	Target or information provided
embB-306	GTCGGACGACGGCTACATCCTGGGCATggcccgagtcgc cgaccacgccggctac	142	EMB resistance marker
katG-315	caccggaaccggtaaggacgcgatcaccaCCGGCATCGAGGTCGT ATGGACGAACACCCC	160	INH resistance marker
inhA-15	CGATTTCGGCCCGGCCGCGGCGAGATgataggttgtcggg gtgactgccacagcc	178	INH resistance marker
16S rRNA	CACGGGATGCATGTCTTGTGGTGGAAAgcgctttagcggt gtgggatgagcccgcggc	202	16S rRNA gene, MTBC specific
rpoB-176	cacgttcatcatcaacgggaccgagcgtgtggtgTTCAGCCAGCTGGT GCGGTCGCCC	229	RIF resistance marker
rpoB-531	GTTGACCCACAAGCGCCGACTGTTggcgctggggcccggcg gtctgtcacgt	256	RIF resistance marker
rpoB-526G	caacccgctgtcggggttgaccGACAAGCGCCGACTGTCGGCG CTGGGGCC	265	RIF resistance marker
rpoB-526T	caacccgctgtcggggttgaccTACAAGCGCCGACTGTCGGCG CTGGGGCC	274	RIF resistance marker
rpoB-522	agccaattcatggaccagaacaacccgctgtTGGGGTTGACCCACA AGCGCCGAC	283	RIF resistance marker
katG-463	GATTGCCAGCCTTAAGAGCCAGATCCGggcatcgggatt gactgtctcacagctagtttcgacc	319	Genotype marker, specific for PGG two and three
gyrA-95	GCACGGCGACGCGTCGATCTACGACACcctggtgcgcat ggcccagccctgg	328	Genotype marker, specific for PGG one and two
mutT2-58	CCCGAGAGCTCGCCGAAGAACTGCgactcgaggtcgccga cctcgcggtggg	355	Genotype marker, specific for Beijing two
mutT4-48	CGACCCCGGCAACGGCGAAGGggtcccggtcccgctcacctcg tcgcgggt	364	Genotype marker, specific for Beijing one, two and three
ogt-12	CGCACCATCGATAGCCCCATCGGAccattaaccctggccggg catggctcggtgttga	373	Genotype marker, specific for Beijing two
ogt-15	taccgcaccatcgatagccccatcgggccattaaGCCTGGCCGGGCAT GGCTCGGTGTTGA	382	Genotype marker, specific for Haarlem
ogt-37	cctgcggatgctcgagcagacgtatgagccaagccTCACACACTGGAC ACCCGACCCC	391	Genotype marker, specific for Beijing three and four
TbD1	GCGGTCGCGGGATTCAGCGTCTATcggttgcacggcatcttc ggctcgcacgaca	418	Absent in modern *Mycobacterium tuberculosis* strains

EMB: ethambutol; INH: isoniazid; RIF: rifampicin; PGG: principal genetic group; MTBC: *Mycobacterium tuberculosis* complex.

*Genotypic characterisation* - Genomic bacterial DNA was extracted from *M. tuberculosis* cultures according to a standard protocol described by van Soolingen et al. (1994). Spoligotyping was performed according to Gomgnimbou et al. (2013), using a microbead-based DNA chip read on a MagPix system (Luminex, Austin, TX). The online database SITVIT WEB (Demay et al. 2012) was used to compare the results with the international shared spoligotypes.

*DNA sequencing procedures* - The key genomic regions involved in the INH resistant phenotype (*kat*G and *inh*A genes) and RIF resistant phenotype (*rpo*B gene) were amplified and sequenced according to protocols previously published (Dalla Costa et al. 2009), respectively. Briefly the sequencing amplifications were carried out in an Applied Biosystems® Veriti® 96-Well thermo cycler (Thermo Fischer Scientific, California, EUA) as follows: 94ºC for 2 min, 55ºC for 1 min, and 72ºC for 2 min, for 30 cycles. Amplification products were analysed by electrophoresis in 1.5% agarose gels, purified PCR products were purified with the polyethylene glycol method and sequenced by using the Big Dye Terminator Cycle Sequencing v 3.1 Kit (Applied Biosystems, Foster City, CA, USA) in the ABI Prism 3130*xl* DNA Sequencer (Applied Biosystems).

*GenoType® MTBDRplus assay* - The Genotype® *MTBDRplus* assay was carried out according to the manufacturer’s instructions [Hain Lifescience, Nehren, Germany, (Crudu et al. 2012)].

*MLPA analysis* - All MLPA reagents were manufactured and supplied by MRC-Holland (Amsterdam, The Netherlands). The fragments were analysed using an ABI 3130xl Genetic Analyzer (Applied Biosystem, Foster City, CA, USA) capillary electrophoresis instrument. The evaluation of these fragments was performed using GeneMapper, software version 3.2 (Applied Biosystem, Foster City, CA, USA).

*Statistical analyses* - Statistical analysis was performed using the SPSS v.21 statistical program (SPSS Ins. Chicago, IL, USA). Agreement between the methods was evaluated using the *kappa* score.

## RESULTS

*Phenotypic Resistance* - From the 96 samples tested, 82 were isoniazid-susceptible (INH-S), 14 were isoniazid-resistant (INH-R), 88 were rifampicin-susceptible (RIF-S) and eight were rifampicin-resistant (RIF-R), by the standard DST performed by the proportion method (Canetti et al. 1969). Associated INH and RIF resistance was found in six samples, defined as MDR-TB strains (Table II).

**TABLE II:** Drug susceptibility testing (DST), sequencing, multiplex ligaton-dependent probe amplification (MLPA) and GenoType MTBDR*plus* results for the 96 isolates studied

N^o^ of samples	DST		Sequencing			GenoType MTBDR*plus* (A)			MLPA (B)		
			
	INH	RIF	*kat*G	*inh*A	*rpo*B	*kat*G	*inh*A	*rpo*B	*kat*G	*inh*A	*rpo*B
79	S	S	WT	WT	WT	WT	WT	WT	WT	WT	WT
4	R	R	S315T	WT	S531L	S315T	WT	S531L	S315T	WT	S531L
4	R	S	S315T	WT	WT	S315T	WT	WT	S315T	WT	WT
1	R	R	S315T	WT	D516V/H526Y/S531L	WT	WT	H526Y/S531L	WT	WT	S531L
1	R	R	S315T	WT	D516V/S531L	S315T	WT	D516V	S315T	WT	WT
1	R	R	S315T	WT	D516V/S531L	S315T	WT	D516V	S315T	WT	S531L
1	R	S	WT	(-15C →T)	WT	WT	WT	WT	WT	(-15C →T)	WT
1	S	R	WT	WT	S531L	WT	WT	S531L	WT	WT	S531L
1	S	S	WT	WT	WT	S315T	WT	WT	WT	WT	WT
1	S	S	WT	WT	WT	S315T	WT	D516V	WT	WT	WT
1	R	S	S315T	WT	WT	S315T	WT	WT	S315T	WT	WT
1	R	S	S315T	WT	WT	S315T	WT	WT	S315T	WT	WT

INH: isoniazid; R: resistant; RIF: rifampicin; S: susceptible; WT: wild-type.

*Relation between phenotypic resistance and mutations in drug-resistance targets for INH and RIF* - From the 14 INH resistant strains characterised by the standard DST test, 13 carried a S315T (AGC-ACC) mutation; one carried an *inh*A -15C →T mutation (Table II). Mutation S531L in the *rpo*B gene was identified in all of phenotypic RIF-resistant samples. From these, three had also a second mutation in the codon D516V (GAC-GTC) and one had the aggregation of a third mutation in codon H526Y (CAC-TAC). All the samples classified as MDR-TB by the phenotypic DST test were confirmed by sequencing (Table II).

*Spoligotyping* - Spoligotyping using the Luminex microbead-based allowed identification of *M. tuberculosis* lineages for 93 of the 96 isolates studied: 58% were Latin American Mediterranean (LAM), 22% Euro American, 15.5% Haarlem, and 2% X lineage. Three of them had indeterminate results. Beijing clade was absent in this collection of samples.

*MLPA* - The identification probe 16SrRNA gave a signal in all samples. The genotype markers probe katG-463 and gyrA-95 also gave a signal in all samples. The TbD1 probe was ligated in most of the samples, indicating that the TbD1 fragment was absent and most strains were “modern” genotypes, as this probe targets the flanking regions of the deleted TbD1 fragment. The *ogt -15* identified all the Haarlem strains that were classified as Haarlem by spoligotyping. No Beijing family strains were identified in this collection of *M. tuberculosis* by MLPA or spoligotyping. Therefore, the sensitivity of probes *ogt*-12, ogt-37, mutT2-58 and mutT4-48 could not be determined. The MLPA embB-306 probe worked in one of the two EMB resistant isolates; previously it was determined that this probe is able to detect only a proportion of the mutations that can arise in the 306 codon of *emb*B. As the *emb*B gene was not sequenced, these results could not be confirmed. Results for INH and RIF related probes are shown below in comparison to the Genotype®*MTBDRplus* assay.

*MLPA x MTB-DR plus* - Both, the MLPA and the Genotype®*MTBDRplus* assay identified MTBC in all samples using the designed MTBC specific probes. Using DST as the reference standard, the specificity of MLPA and Genotype®*MTBDRplus* for detection of INH resistance was 100% [95% confidence interval (CI): 95-100] and 97.5% (95% CI: 91-99), respectively; for detection of RIF resistance was 100.0% (95% CI: 99-100) and 98.8% (96-100), respectively. The MLPA and Genotype®*MTBDRplus* sensitivity was 92.8% (95% CI: 66-99) and 85.7% (95% CI: 59-97), respectively, to detect INH resistance; and 87.5% (95% CI: 51-99) and 100%, (95% CI: 63-100) to detect RIF resistance (Table III). Assuming sequencing as reference standard for the genotypic characterisations by the MLPA and Genotype®*MTBDRplus* methods, the sensitivity to INH resistance detection was 92.8%, (CI 95% 66-99) and 85.7% (CI 95% 59-97) respectively, and the specificity was 100% (CI 95% 95-100) and 97.5% (CI 95% 91-99), respectively. In relation to RIF resistance, the sensitivity was 85.7% (CI 95% 51-99) and 100% (CI 95% 63-100), respectively, and the specificity was 100% (CI 95% 99-100) and 98.8% (CI 95% 96-100), respectively (Table IV). The agreement, measured by kappa (k) between MLPA and sequencing was the same to MLPA and DST: 0.95 (95% CI: 0.90 - 99) for INH and 0.92 (95% CI: 0.86 - 0.97) for RIF, respectively. As well as, Genotype®*MTBDRplus* showed the same kappa results when it was compared to sequencing and to DST: K = 0.83 (95% CI: 0.74 - 0.91) to INH and 0.93 (95% CI: 0.87 - 0.98) to RIF (Tables III, IV).

**TABLE III:** Multiplex ligaton-dependent probe amplification (MLPA) x GenoType MTBDR*plus* in comparison to drug susceptibility testing (DST) and sequencing for the 96 isolates of *Mycobacterium tuberculosis* included in the study

Methods	INH		Assessment characteristic (%)			RIF		Assessment characteristic (%)		
	
	Nº of strains (%)					Nº of strains (%)				

	Resistant (n = 14)	Susceptible (n = 82)	Sensitivity	Specificity	Kappa (95% CI )	Resistant (n = 8)	Susceptible (n = 88)	Sensitivity	Specificity	Kappa (95% CI)
MTBDR*plus* (A)										
Mutant	12	2	85.7 (59 - 97)	97.5 (91 - 99)	0.83 (0.75 - 0.91)	8	1	100 (63 - 100)	98.8 (96 - 100)	0.93 (0.88 - 0.98)
WT	2	80				0	87			
MLPA (B)										
Mutant	13	0	92.8 (66 - 99)	100 (95 - 100)	0.95 (0.91 - 0.100)	7	0	85.7 (51 - 99)	100 (99 - 100)	0.92 (0.87 - 0.98)
WT	1	82				1	88			

INH: isoniazid; RIF: rifampicin; WT: wild-type.

**TABLE IV:** Multiplex ligaton-dependent probe amplification (MLPA) vs MTBDR*plus* in comparison to sequencing for assessment sensitivity, specificity and agreement of both methods

Methods	INH		Assessment characteristic (%)			RIF		Assessment characteristic (%)		
	
	Nº of strains (%)					Nº of strains (%)				

	Resistant (n = 14)	Susceptible (n = 82)	Sensitivity (95% CI)	Specificity (95% CI)	Kappa (95% CI)	Resistant (n = 8)	Susceptible (n = 88)	Sensitivity (95% CI)	Specificity (95% CI)	Kappa (95% CI)
MTBDR*plus* (A)										
Mutant	12	2	85.7 (59 - 97)	97.5 (91 - 99)	0.83 (0.75 - 0.91)	8	1	100 (63 - 100)	98.8 (96 - 100)	0.93 (0.88 - 0.98)
WT	2	80				0	87			
MLPA (B)										
Mutant	13	0	92.8 (66 - 99)	100 (95 - 100)	0.95 (0.91 - 1.00)	7	0	87.5 (51 - 99)	100 (99 - 100)	0.92 (0.87 - 0.98)
WT	1	82				1	88			

INH: isoniazid; RIF: rifampicin; WT: wild-type.

## DISCUSSION

The currently available methods to identify *M. tuberculosis* and mutations related to drug resistance are mainly based on the PCR amplification of target genes or mutations and subsequent analysis of the amplified product by reverse or direct hybridisation with complementary probes, sequencing or melting point analysis. As a limitation, these methods do not allow multiplexing of many targets (the exact number depends on the thermodynamic and structural characteristics of the sequences to be amplified) without a significant loss of specificity and overall accuracy (Bergval et al. 2008). An efficient and high-multiplex assay, able to detect several widely dispersed point of mutations and gene sequences involved in tuberculosis drug resistance and molecular typing, especially for complicated cases of TB recurrence, could benefit patient treatment and would be a very useful tool for the management of efficient TB control programs. Genotyping allows distinguishing between recurrent cases of reinfection or reactivation (Unis et al. 2014), enabling molecular epidemiological studies to monitor the genotypic diversity of TB, as some particular genotypes are correlated with disease dynamics, outbreak detection and increased virulence (Narvskaya et al. 2002, Gomes et al. 2012, Perizzolo et al. 2012, Dalla Costa et al. 2015).

Unless isolates are routinely typed it will not be possible to further explore these associations. Moreover, clinically severe cases of TB are more prevalent among presumed extensively drug-resistant TB (XDR-TB) cases, treatment dropout, and/or TB and M/XDR-TB outbreaks.

The MLPA genotype marker probes katG-463 and gyrA-95, specific for principal genetic group (PGG) one, two and three families, allow the discrimination between the different bacterial subspecies (Bergval et al. 2008) (Table I). These results highlight the potential use of MLPA, as it appears ligated in all samples; however there were some interpretation concerns. Further studies focusing on phylogenic view should be conducted to better understand these results. TbD1 probe was ligated in most of the samples, this probe targets the TbD1 deletion site and thus the presence of its product indicates the presence of the TbD1 deletion. As the TbD1 probe region is absent in modern *M. tuberculosis* strains (Baker et al. 2004), it was possible to infer that most of the tested samples are so called “modern” strains. Additionally, the inclusion of only two *M. tuberculosis* isolates resistant to EMB precluded the evaluation of the EMB drug resistance performance of MLPA. However, as the embB-306 probe has previously been shown to be specific for only two out of the three possible mutations in this codon, absence of a mutation cannot be concluded from absence of an MLPA product (Sreevatsan et al. 1997).

Genotype®*MTBDRplus* has been compared to phenotypic and other molecular assays with very impressive performance and was endorsed by WHO for MDR-TB molecular detection in 2008 (Nikolayevsky et al. 2009, Ferreira Jr et al. 2014, Asante-Poku et al. 2015). Since the first report using MLPA as a promising alternative for TB genetic characterisation (Bergval et al. 2008), few studies evaluating MLPA in comparison to other assays have been described (Bergval et al. 2012, Sengstake et al. 2014, Chaidir et al. 2016). In this scenario, as EMB and PZA susceptibility testing show no reliable results, and having small sample size of drug-resistant *M. tuberculosis* strains, in our study we relied on the evaluation of MLPA performance on RIF and INH resistance.

The concordance between results of MLPA and sequencing (gold-standard) was slightly higher than Genotype®*MTBDRplus* versus sequencing for the assessment of INH resistance. Asante-Poku et al. (2015) reported misdiagnosis by Genotype®*MTBDRplus* in approximately 16% of INH-monoresistant isolates that were wrongly identified as susceptible, reinforcing the possibility that Genotype®*MTBDRplus* may have limitations in the detection of INH resistance, depending on the local prevalence of unusual mutations. Simons et al. (2015) also reported low sensitivity (36%) of the Genotype®*MTBDRplus* in detecting *inh*A gene mutations.

Regarding the agreement between Genotype® *MTBDRplus* and MLPA versus the standard DST (Table III) and sequencing (Table IV), results were very concordant for INH (Kappa A = 0.83 and kappa B = 0.95) and RIF (Kappa A = 0.93 and kappa B = 0.92) resistance detection with a better performance of the MLPA assay since it was able to correctly detect the *inh*A (-15C →T) mutation responsible for the INH resistance detected by the DST assay. Concerning the overall distribution of mutations associated with INH resistance, the most frequent mutation was *kat*G S315T, similarly to what has been observed by other authors (Dalla Costa et al. 2009, Cambau et al. 2015).

The S531L mutation in the *rpo*B gene was the most frequent mutation found in the RIF resistant isolates, followed by mutations in codon D516V, similar to that reported elsewhere (Perizzolo et al. 2012, Maschmann et al. 2013). For the detection of RIF the discrepant results occurred with three samples; Genotype®*MTBDRplus* detected one D516V *rpo*B mutation in a sample with no mutation in the *rpo*B region. Drobniewski et al. (2012) reported that both Genotype®*MTBDRplus* and GeneXpert MTB/RIF assays may present lower specificity for RIF resistance detection. MLPA was unable to detect *rpo*B mutations in one sample, maybe due to the presence of strains exhibiting heteroresistance as described by other researchers on *rpo*B, D516V/S531L (Nikolayevsky et al. 2009, van Deun et al. 2009) (Table I). Genotype®*MTBDRplus* correctly determined RIFr but only detected the D516V mutation, the S531L was not detected (Table II).

MLPA allowed the identification of all the Haarlem genetic clade strains with the ogt-15 probe, but this test did not cover the T and LAM prevalent lineages of *M. tuberculosis* in Brazil, as described elsewhere (Gomes et al. 2012). Beijing probes are also included in this MLPA version, no ligation of these probes was seen in any isolate, in agreement with spoligotyping results.

The MLPA and Genotype®*MTBDRplus* specificity for detecting INH resistance was 100.0% and 97.5% respectively; and 100.0% and 98.8% respectively for detection of RIF resistance. MLPA and Genotype®*MTBDRplus* showed higher agreement with sequencing than DST. Overall, the MLPA performance to detect drug-resistant TB and MDR-TB showed similar accuracy to Genotype®*MTBDRplus*, with the advantage of being easily optimised on a routine basis to address accurately the early detection of MDR-TB, with the possibility to expand to further drug targets and antibiotics.

In comparison to other molecular assays, MLPA is a test that allows adjustments in probe composition, thus targeted loci can be adapted to the local prevalence of certain mutations by a simple addition or removal of probes from the master mix. As a final consideration regarding study limitations, a limited number of clinical isolates with INH and/or RIF resistance was evaluated and minimal inhibitory concentration (MIC) was not performed. However, we observed that MLPA, and other truly multiplexed assays adapted to tuberculosis diagnosis, can be particularly suitable for detecting additional drug resistance to other antimicrobials in a single step, being especially important in recurrence, treatment dropout, or presumed XDR-TB for a rapid and proper treatment decision. Moreover, MLPA may allow complementation to the diagnosis with epidemiological *M. tuberculosis* lineages information to monitor the DR/MDR-TB burden and evolution in different regions, especially in regions where routine drug susceptibility testing is not implemented in laboratories and where the rates of M/XDR-TB is suspected to be high. The flexibility and specificity of MLPA, demonstrated above, along with its ability to simultaneously genotype and detect drug resistance mutations, make MLPA an easy to implement and very important molecular tool for increased drug resistance coverage and early detection of M/XDR-TB as recommended by WHO and the Brazilian Ministry of Health (MS 2010, WHO 2015).

*Ethics approval* - The study was approved by the research ethics committee of UFRJ, ruling number P 11821913.6.0000.5257.
